# A Case Report Demonstrating the Favorable Outcomes of Using N-acetylcysteine (NAC) in Managing Hepatic Injury Induced by Amphetamine-Related Drug Toxicity: Do We Underestimate Its Potential?

**DOI:** 10.7759/cureus.53697

**Published:** 2024-02-06

**Authors:** Gagan Aulakh, Arshdeep Singh

**Affiliations:** 1 Internal Medicine, Jersey City Medical Center, Jersey City, USA; 2 Internal Medicine, Government Medical College, Amritsar, Amritsar, IND

**Keywords:** n-acetylcysteine, drug toxicity, methampheatmine, drug-induced acute liver failure, acute liver failure

## Abstract

A 59-year-old male with a history of alcohol abuse presented with altered mental status. Upon examination, he was hypertensive and lethargic, and laboratory results revealed severe transaminitis, coagulopathy, and lactic acidosis, despite having normal serum alcohol levels. Additionally, his urine drug screen tested positive for methamphetamine. Following the exclusion of infectious, autoimmune, and other common causes of acute hepatitis, a diagnosis of methamphetamine-induced acute hepatitis was established. A non-acetaminophen toxicity N-acetylcysteine (NAC) protocol was initiated, resulting in a positive response with improvement in mentation and a decrease in liver enzyme levels. This case emphasizes the potential effectiveness of NAC in treating amphetamine-induced liver injury, supported by the limited available literature on the subject.

## Introduction

Acute hepatitis is characterized by damage to the hepatic parenchyma occurring within six weeks, leading to liver dysfunction. If the condition persists beyond six weeks, it is termed chronic hepatitis. Methamphetamine is classified within the ischemic category for its association with hepatitis [1]. Acute liver failure (ALF) is diagnosed when a patient, without any underlying liver disease, develops encephalopathy and coagulopathy, with an international normalized ratio (INR) greater than 1.5, in a period of less than 26 weeks [2]. Viral and drug-induced acute liver injury are the most common causes; however, other potential factors include ischemic events, Budd-Chiari syndrome, autoimmune diseases, Wilson's disease, mushroom toxicity, and others. In the United States, among drug-induced causes, acetaminophen-induced ALF is the most common, for which N-acetylcysteine (NAC) is the drug of choice along with the management of complications and possible liver transplant [2,3]. In Spain, hepatitis secondary to amphetamine use ranks as the second most common form of hepatitis in individuals under the age of 25, following viral hepatitis [4].

Liver toxicity induced by amphetamine typically manifests three to 14 days after ingestion, and the underlying pathophysiology remains unclear, giving rise to multiple hypotheses. Various hypotheses of liver injury mechanisms by amphetamines are, including but not limited to, injury caused by toxic metabolites generated by hepatic cytochrome P450 during the metabolism of high-dose amphetamines, a deficiency of cytochrome P450 in 5-9% of Caucasians, immune-mediated mechanisms, and hepatic injury related to hyperthermia [4,5]. Microscopically, amphetamine-induced hepatotoxicity may reveal microvascular fatty changes or hepatic necrosis [4]. NAC aids in reducing oxidative stress by diminishing free radicals through the replenishment of intra-hepatocyte glutathione. Additionally, it promotes an increase in nitrite levels, leading to vasodilation, thereby enhancing oxygen delivery to tissues. [3].

## Case presentation

A 59-year-old male was brought to the emergency room after being discovered unconscious in the field. He had a medical history of coronary artery disease treated with coronary artery bypass grafting (CABG), chronic kidney disease (CKD), heart failure characterized by a reduced ejection fraction of 30%, diabetes, hypertension, alcohol abuse, and non-compliance with medications. A point-of-care glucose check indicated a blood glucose level below 50 mg/dL, prompting the administration of dextrose pushes, which resulted in a slight improvement in his mental state. His examination was unremarkable except for icterus and lethargy. His vital signs showed a blood pressure (BP) of 180/97mmHg, a heart rate (HR) of 110 bpm, a respiratory rate (RR) of 30 breaths per minute, and saturation of peripheral oxygen (SpO2) of 97% on room air. Further assessment revealed the laboratory values as given in Table 1, and a urine drug screen tested positive for amphetamines. The CT head scan showed no evidence of acute pathology.

**Table 1:** Blood workup at admission The baseline levels of AST, ALT, ALP, bilirubin, BUN, and creatinine are 20 U/L, 30 U/L, 100 U/L, 1.1 mg/dL, 40 mg/dL, and 2 mg/dL, respectively.

Test	Result	Reference value
Aspartate transaminase (AST) U/L	5086	8-33
Alanine transaminase (ALT) U/L	1613	4-36
Alkaline phosphatase (ALP) U/L	136	33-96
International normalized ratio (INR)	2.5	<1.1
Total bilirubin (mg/dL)	3.9	0.1-1.2
Creatine phosphokinase (CPK) U/L	4000	46-171
Ammonia (umol/L)	31	11-31
Albumin (g/dL)	4	3.2-4.8
Lactic acid (mmol/L)	8.8	0.5-1.99
Anion gap (mmol/L)	19	< 18
Serum bicarbonate (mmol/L)	15	20-30
Serum alcohol (mg/dL)	< 3	<10
Serum acetaminophen (mcg/mL)	2.4	10-20
Serum salicylate (mg/dL)	6.5	<29
Platelet count	50,000/mcL	130,000-400,000/mcL
Blood urea nitrogen (BUN) (mg/dL)	74	9-23
Creatinine (mg/dL)	3.77	0.6-1.2

At this time, differentials were viral or drug-induced hepatitis. He was admitted to the ICU; the diagnosis included acute liver injury accompanied by rhabdomyolysis and acute kidney injury (AKI) superimposed on CKD. The arterial blood gas analysis revealed metabolic acidosis with respiratory compensation, indicating a pH of 7.30, pCO2 of 27 mmHg (reference 35-45), and bicarbonate level of 13.3 mmol/L (reference 22-28). The workup for other infectious and autoimmune conditions was negative as detailed in Table 2. An ultrasound liver test with Doppler was normal and ruled out a thrombus. Following consultation with nephrology, the patient was initiated on a dextrose 5% bicarbonate 150mEq drip at 150mL/hr after a 1-liter normal saline bolus. On day two, the transaminitis worsened as indicated in Table 3. After ruling out all the causes, a methamphetamine-induced liver toxicity diagnosis was made. Then the patient was started on NAC protocol for 48 hours.

**Table 2:** Infectious and autoimmune blood workup at admission

Test	Result
Hepatitis A IgM antibody	Non-reactive
Hepatitis B surface antigen	Non-reactive
Hepatitis B core antibody	Non-reactive
Hepatitis C virus antibody	Non-reactive
Cytomegalovirus IgM antibody	Not detected
Epstein-Barr virus IgM antibody	Negative
Herpes simplex virus type 1	Negative
Herpes simplex virus type 2	Negative
Varicella IgM antibody	Negative
Antinuclear antibody (ANA)	Negative
Smooth muscle antibody, qualitative titer	Negative

**Table 3:** Daywise blood workup

Test	Day two	Day three	Day four	Day five	Day six
Aspartate transaminase (AST) (U/L)	>6000	4057	3711	2469	1189
Alanine transaminase (ALT) (U/L)	2093	1579	1057	770	553
International normalized ratio (INR) (U/L)	2.42	2.21	1.55	1.31	1.27
Bilirubin (mg/dL)	3.4	2.4	2.6	2.1	2.0

The NAC protocol involved a loading dose of 150 mg/kg administered over one hour, followed by 50 mg/kg for the subsequent four hours, and a maintenance infusion at a rate of 6.25 mg/kg/hr. Following the administration of NAC, there was an improvement observed in the patient's mental status, along with positive changes in liver functions. Our patient remained hemodynamically stable and afebrile throughout the entire duration of the treatment and was then transferred to routine medical floors, which was followed by a discharge home.

## Discussion

To the best of our knowledge, there is only one reported case documenting the use of NAC in the context of amphetamine-induced liver injury. This information was obtained through a PubMed search using the terms "N-acetylcysteine" and "Amphetamine" for the period from 1992 to 2024 [6]. Affas et al. employed NAC in the context of amphetamine-induced liver injury; however, in our case, there are significantly elevated transaminase levels exceeding 6000 U/L compared to the reported 2500 U/L. Furthermore, our patient remained hemodynamically stable throughout, eliminating the possibility of shock liver related to hypotension [6]. We attributed lactic acidosis to reduced liver clearance. In an in-vitro experiment, Zhang et al. utilized immortalized human brain endothelial cells, pre-treating them with NAC before exposing them to methamphetamine. Their observations revealed a reduction in the production of oxidative stress byproducts, such as reactive oxygen species, and byproducts of lipid peroxidation like malondialdehyde. Additionally, there was an augmentation in the concentration of intracellular glutathione and glutathione peroxidase [7]. There have been successful trials demonstrating the efficacy of NAC in the treatment of amphetamine dependency [8]. Parvataneni et al. employed NAC in shock liver, contrary to the belief that NAC should not be used for shock or ischemic hepatitis. They observed positive results, and the use of NAC helped prevent the need for liver transplant [1,3].

A limitation in our case report is the unknown dose of amphetamine taken by our patient.

## Conclusions

NAC demonstrates a favorable response in cases of methamphetamine-induced liver injury, effectively preventing the progression of liver damage. Further research on the use of NAC for amphetamine-induced liver injury is warranted, and its consideration should be made without hesitation if deemed necessary. Physicians are hesitant to use it due to the absence of guidelines. Our patient exhibited a positive and highly effective response to NAC.
